# Longitudinal Coupling between Eating Disorder Psychopathology and Depression in Patients with Anorexia Nervosa and Bulimia Nervosa Treated with Enhanced Cognitive Behavior Therapy: A One-Year Follow-Up Study

**DOI:** 10.3390/brainsci13040535

**Published:** 2023-03-24

**Authors:** Emanuele Cassioli, Eleonora Rossi, Michela Martelli, Francesca Arganini, Gabriele Giuranno, Serena Siviglia, Livio Tarchi, Marco Faldi, Giovanni Castellini, Valdo Ricca

**Affiliations:** Psychiatry Unit, Department of Health Sciences, University of Florence, Largo Brambilla 3, 50100 Florence, Italy; emanuele.cassioli@unifi.it (E.C.);

**Keywords:** anorexia nervosa, bulimia nervosa, eating disorder, depression, enhanced cognitive behavior therapy, structural equation model

## Abstract

Background: The relationship between eating disorder (ED) specific psychopathology and depressive symptomatology in EDs is often debated. The aim of this study was to provide an explicative model regarding the mechanisms by which enhanced cognitive-behavior therapy (CBT-E) might determine an amelioration of depressive symptoms in patients with anorexia nervosa (AN) or bulimia nervosa (BN). Methods: A total of 157 women with AN or BN and no history of childhood trauma or bipolar disorder were evaluated before treatment and after 12 months of CBT-E. Self-administered questionnaires were used to measure ED psychopathology and depressive symptoms. Results: All psychopathological measures improved after treatment, with no significant additional improvement with the concomitant use of antidepressants. Structural equation modeling using the bivariate latent change score approach showed that higher levels of depressive symptoms at baseline were associated with a worse longitudinal trend of ED psychopathology, and vice versa. Finally, the amelioration of ED psychopathology predicted the improvement in depressive symptoms at follow-up, whereas data did not support the inverse path. Conclusion: This study elucidated the complex longitudinal interplay between ED psychopathology and depression during CBT-E, underlining the importance of addressing ED symptoms as a primary target in the case of comorbidity between AN or BN and depressive symptoms.

## 1. Introduction

Anorexia nervosa (AN) and bulimia nervosa (BN) are severe eating disorders (EDs) that share a common psychopathological core represented by the excessive importance attributed to body shape and weight in determining one’s own self-esteem [1,2]. Individuals with AN and BN show high rates of comorbidity with other psychiatric conditions [3,4]. In particular, major depressive disorder, with a prevalence exceeding 40% in these patients, is considered among the most frequent co-occurring diagnosis [3,4]. Comorbid depression has been associated with worse ED outcomes [5,6,7], a greater risk of suicidal behaviors [8,9,10,11], and a greater likelihood of diagnostic crossover [12]. Furthermore, recent studies based on network analysis demonstrated the centrality of affective problems as the core psychopathological features of EDs [13,14]. From a qualitative point of view, Voderholzer et al. [15] showed that patients with EDs had similar core aspects of depression compared with patients with a primary diagnosis of depressive disorders, such as sadness and loss of pleasure, and more pronounced symptoms referring to the area of negative self-view. Several authors tried to explain the relationship between depressive symptoms and ED psychopathology, suggesting that depression may precede the onset of EDs, which would be sequelae of affective problems, whereas, on the contrary, others hypothesized that depression might be a consequence of the ED [16]. Furthermore, depressive symptoms could be the result of the malnutrition status that is often associated with EDs, resulting from pathological eating behaviors [2].

Regarding the psychopathology of depression, patients with specific comorbidities deserve a separate discussion. First, depressive symptoms in the context of a comorbid bipolar disorder might represent the epiphenomenon of a specific mood instability resulting from a biological vulnerability [2,17,18,19,20]. Moreover, it appears that in the subgroup of patients with childhood trauma the network structure of ED symptoms, specifically relating to depressive mood, might be different [21]. In particular, depressed mood was found to be a central and driving symptom in patients with EDs and a history of childhood abuse. On the contrary, depressed mood emerged as a consequence of specific characteristics of the ED in patients reporting no exposure to traumatic experiences during childhood [21].

Despite being among the first-line treatments for AN and BN, enhanced cognitive-behavior therapy (CBT-E) [1,22] is considered contra-indicated in the case of comorbidity of the ED with severe depression [1]. In particular, cognitive-behavioral theorists recommend that clinical depression should be treated with full-dose antidepressants before initiating the psychotherapeutic treatment [1]. However, treatment with antidepressants did not prove to be efficacious in alleviating depression in patients with AN or BN [23,24], and it has been theorized that it might be preferable to focus on ED symptoms when treating these patients, regardless of the presence of clinical depression [25,26]. Consistent with this hypothesis, Calugi et al. [27] showed that, in a sample of patients with AN, both ED psychopathology and depressive symptoms improved after treatment with CBT-E alone, both in patients with and without a diagnosis of clinical depression at baseline. However, to the best of our knowledge, at present no study provides an explicative model of the mechanisms by which CBT-E could determine an amelioration of depressive symptoms in patients with EDs. Therefore, it is still controversial whether the improvement in depression is an effect of the amelioration of ED symptoms or vice versa, and it is not clear which symptoms should be primarily targeted in the case of comorbidity between AN or BN and depressive symptoms.

According to the transdiagnostic cognitive-behavioral model of EDs, the overvaluation of one’s ability to control body shapes and weight as the only way to determine one’s own self-esteem is a crucial factor in exacerbating the marginalization of other areas of life [1]. Therefore, this core psychopathological nucleus is involved in determining a progressive impoverishment of the social functioning of patients with EDs [1], which is a very well-known risk factor for the development and maintenance of depressive symptoms [28,29]. Furthermore, overinvesting in one single area for the determination of one’s self-esteem exposes one to feelings of failure and low self-efficacy [1], and the persistence of the ED-specific psychopathological core has been associated with the maintenance of negative body image [30,31], which is deeply interconnected with negative affect in patients with EDs [32,33,34]. 

In light of these considerations, it could be hypothesized that the improvement in depressive symptoms in patients with AN or BN necessarily requires the erosion of the ED-specific psychopathological core, and that CBT-E might determine an improvement in depressive symptoms through its action on this nucleus. Considering the multifaceted psychopathological meaning of depression in different subpopulations of patients with EDs, as previously mentioned, specifically those with comorbid bipolar disorder or a history of childhood trauma, this study aimed to test this hypothesis in a sample that was as homogeneous as possible to avoid potentially misleading generalizations. Therefore, the direction of the longitudinal coupling between the variation over time in depressive symptoms and ED-psychopathology was explored through the application of the latent change score (LCS) technique in the context of structural equation modeling (SEM) analysis in a sample of patients with AN or BN without a history of bipolar disorder or severe childhood trauma. 

## 2. Materials and Methods

This was a longitudinal observational study with a one-year follow-up. All participants received adequate information about the study procedures and signed informed consent. The ethics committee of the local institution approved the study protocol, which was carried out according to the guidelines of the Declaration of Helsinki of 1964 and subsequent amendments. 

### 2.1. Participants 

Patients who performed the first clinical evaluation at the Clinic for EDs of the University of Florence between June 2018 and March 2021 were enrolled in the present study, provided they met the following inclusion criteria: female sex; age between 18 and 60 years; current diagnosis of AN or BN according to the Diagnostic and Statistical Manual of Mental Disorders—Fifth Edition (DSM-5) [2], as assessed by the Structured Clinical Interview for DSM-5 Disorders, clinical version (SCID-5-CV) [35]. The following exclusion criteria were applied: diagnosis of comorbid bipolar disorder or psychotic disorder defined according to DSM-5 [2], as assessed by SCID-5-CV; severe medical (e.g., cardiac or renal failure) or psychiatric (e.g., severe suicidal ideation) conditions precluding outpatient treatment; treatment with psychotropic medication, except for antidepressants and benzodiazepines; history of severe childhood trauma; intellectual disability; illiteracy or any other condition that could compromise the understanding of the protocol and the completion of the questionnaires; absence of written informed consent. The history of severe childhood trauma, as defined by the World Health Organization [36], was evaluated through a clinical interview and using the validated cut-off values of the Childhood Trauma Questionnaire [37], which are the following: ≥16 for emotional abuse, ≥13 for physical abuse, ≥13 for sexual abuse, ≥18 for emotional neglect, ≥13 for physical neglect.

Of the 210 patients with AN or BN who were consecutively referred, 12 individuals declined to participate, and 41 met exclusion criteria. 

### 2.2. Assessment

The assessment was performed by psychiatrists with expertise in diagnosing and treating EDs both at baseline (T0) and one year after the enrolment (T1), and included the collection of sociodemographic and clinical data through a face-to-face interview, and anthropometric measures through standard calibrated instruments. Body Mass Index (BMI) was calculated as weight in kilograms divided by the square of height in meters. Furthermore, all patients completed the following self-administered questionnaires in their validated Italian version: 

The Eating Disorder Examination Questionnaire 6.0 (EDE-Q) [1,38], for the evaluation of ED-specific psychopathology through four subscales concerning four core features of EDs (dietary restraint, eating concern, weight concern, and shape concern) and a total score (Cronbach’s α in the present sample = 0.95). An empirically validated cut-off value of 2.5 for the total score can be used in order to distinguish between patients and controls (Rø, Ø., Reas, D. L., and Stedal, K., 2015) [39].

The Beck Depression Inventory Second Edition (BDI-II) [40,41], for the evaluation of depressive symptoms. This provides a total score that can be obtained by summing the scores of each of the 21 items (Cronbach’s α in the present sample = 0.91). A score of 20 or higher is commonly used to identify patients at risk for moderate depression, whereas a score of 29 or higher indicates severe depression. 

The Symptom Checklist-90-Revised [42,43], which assesses general psychopathology through a Global Severity Index (GSI), obtainable by averaging the scores of all items (Cronbach’s α in the present sample = 0.98).

### 2.3. Treatment

All patients were treated in a multidisciplinary setting, including regular psychiatric and dietetic evaluations and, if required, internal medicine visits. Furthermore, they received at least 40 individual CBT-E sessions [1], which were initially administered weekly or twice a week. In the last treatment phase, sessions were scheduled every two or three weeks, depending on individual needs. Weekly multidisciplinary meetings were held to monitor the correct treatment implementation. The median number of CBT-E sessions administered to each participant was 43 (range: 40–52). Furthermore, based on clinical judgment, patients with major depressive disorder according to DSM-5 criteria [2] were treated with selective serotonin reuptake inhibitor (SSRI) antidepressants, in accordance with the current guidelines [44]. These interventions were part of the clinical routine and were not influenced by the enrolment in the present study.

### 2.4. Statistics

Sample data are reported as means and standard deviations. A comparison of baseline data was performed between patients who also completed the second assessment and those who did not, using age- and BMI-adjusted Analysis of Covariance (ANCOVA). Longitudinal changes between T0 and T1 evaluations were tested using age- and BMI-adjusted linear mixed models with random intercepts, with time as a fixed effect. Change in BMI over time was analyzed separately for the two diagnostic groups (AN and BN), using simple slopes analysis in the context of a moderated linear regression. Additionally, similar moderation analyses were carried out to verify whether antidepressant treatment was associated with different outcomes, thus representing a possible confounding effect with respect to the primary objective of the study. The same approach was also used to verify that the trend over time of the patients under treatment was similar in the two diagnostic groups (AN and BN), so as to justify the use of the entire sample for subsequent analyses, in accordance with the transdiagnostic model.

To investigate the complex longitudinal interplay between the psychopathological domains of ED-related psychopathology and depressive symptoms, the LCS technique was used in the context of SEM analysis [45]. With this particular method, it is possible to fix regression coefficients and factor loadings in such a way as to capture the longitudinal variation of a variable from one timepoint to the next in a latent variable, the LCS. With this setting and by entering two different domains with their variations over time in the same model (bivariate LCS model), repeated-measures analyses can be carried out taking into account different effects simultaneously: the constant variation over time (which corresponds to the intercept of the LCS, “α”), the longitudinal variation proportional to the previous timepoint (proportional or autoregressive effect, “β”), the longitudinal variation proportional to the previous timepoint of the other domain (cross-lagged effect, “γ”), and the longitudinal variation proportional to the variation in the other domain (“ε”).

For the present study, a bivariate LCS model was hypothesized, with longitudinal variations in ED psychopathology (ΔEDE-Q) and depressive symptoms (ΔBDI). Baseline EDE-Q and BDI scores were allowed to covary. The following equations were entered:

ΔEDE-Q = α_EDE-Q_ + β_EDE-Q_⋅EDE-Q_T0_ + γ_EDE-Q_⋅BDI_T0_ + ε_EDE-Q_⋅ΔBDI (1)

ΔBDI = α_BDI_ + β_BDI_⋅BDI_T0_ + γ_BDI_⋅EDE-Q_T0_ + ε_BDI_⋅ΔEDE-Q (2)

Cov(EDE-Q_T0_, BDI_T0_) (3)

For the initial model, both LCS intercepts were fixed to zero. Moreover, since the a priori hypothesis predicted that the improvement over time in depressive symptoms was secondary to that of ED psychopathology, but not vice versa, the ε_EDE-Q_ effect was also initially fixed to zero. All these assumptions were tested using nested model comparisons, a technique that allows comparing a hypothesized model with an alternative one in which a previously fixed effect is instead freely estimated (unconstrained model): a statistically significant χ^2^ difference test suggests that the additional parameter significantly improves the model; otherwise the effect does not improve the model and can be kept fixed to zero [46]. The same technique was used to investigate whether the initially hypothesized and freely estimated effects were supported by data, by comparing the initial model with an alternative one in which one of these effects is instead fixed to zero (constrained model).

Heteroskedasticity-robust standard errors were computed for all SEM analyses using the Huber–White sandwich estimator. To facilitate model convergence, BDI scores were divided by 10: regression coefficients and variances should be interpreted accordingly. All models were investigated on the whole sample of patients using the full-information maximum likelihood (FIML) method [47]. Model–data fit was tested by computing the robust version of the following goodness-of-fit indices [48]: χ^2^ test (should be above the threshold of statistical significance, as it assesses the discrepancy between observed and fitted covariance matrices), Comparative Fit Index (CFI, ≥0.95 for good fit), Tucker–Lewis Index (TLI, ≥0.95 for good fit), Root Mean Square Error of Approximation (RMSEA, ≤0.06 for good fit), Standardized Root Mean Square Residual (SRMR, ≤0.08 for good fit). The Bayesian Information Criterion (BIC) was also computed for all models, which is based on the discrepancy between observed and predicted values, as a function of the number of parameters, and allows for an objectively defined selection between different models. Therefore, the lower the BIC, the better the model, as BIC is also a measure of error variance. In comparison to other information criteria, BIC penalizes models with more parameters more severely, allowing for a robust interpretation of results [49].

All analyses were performed using R Statistical Software version 4.1.2 [50] and the following packages: dplyr [51], lavaan [52], nlme [53]. 

## 3. Results

The final sample consisted of 157 participants suffering from EDs, of which 92 (58.6%) had AN and 65 (41.4%) BN. Of the initial sample, 121 (77.1%) patients completed treatment and were evaluated again after 12 months. Compared to these patients, those who had dropped out of treatment or did not complete the follow-up visit showed lower baseline EDE-Q Restraint scores (2.81 ± 2.07 vs. 3.64 ± 1.77, *p* = 0.020); no other differences were found between these groups.

The overall characteristics of the sample at baseline and follow-up evaluations are reported in Table 1. There was a statistically significant improvement in all psychopathological measures after 12 months of treatment (Table 1). More than half of the participants who completed the treatment showed an EDE-Q Total Score below the clinical cut-off of 2.5 at follow-up (*n* = 65 [53.7%]), and the majority also had a BDI score below the threshold corresponding to moderate depression (*n* = 90 [74.4%]). Regarding BMI, patients with AN showed a marked difference at follow-up (BMI_T0_ = 16.34 ± 1.43, BMI_T1_ = 17.59 ± 2.84, *p* < 0.001), whereas patients with BN showed no significant change (BMI_T0_ = 23.69 ± 6.56, BMI_T1_ = 22.48 ± 5.16, *p* = 0.166). No difference was found between AN and BN in the longitudinal trend of all psychometric measurements, as shown by the non-statistically significant interaction effects reported in Supplementary Table S1.

Although a total of 93 (59.2%) participants received a diagnosis of major depressive disorder at the time of the initial assessment, only 32 (20.4%) exhibited severe symptoms (with BDI scores of 29 or higher) and consequently received treatment with antidepressants (SSRIs) in addition to psychotherapy (n_fluoxetine_ = 11; n_sertraline_ = 9; n_escitalopram_ = 5; n_paroxetine_ = 4; n_citalopram_ = 3). These patients showed improvements similar to those of patients without drug therapy, as indicated by moderation analyses, in both depressive symptoms (b_Time⋅Group_ = −2.20, *p* = 0.381) and ED psychopathology (b_Time⋅Group_ = −0.24, *p* = 0.468).

### Structural Equation Model

The proposed model showed excellent model–data fit indices (see ‘Final Model’, Table 2). All variations of this model were tested using nested model comparisons. Analyses involving unconstrained models did not support the addition of LCS intercepts or the effect of ΔEDE on ΔBDI (ε_EDE-Q_) to the model, as the estimation of these coefficients did not significantly improve the initial model (see ‘Unconstrained Models’, Table 2). Conversely, all initially hypothesized effects significantly improved the model and were therefore retained (see ‘Constrained Models’, Table 2).

**Figure 1:**
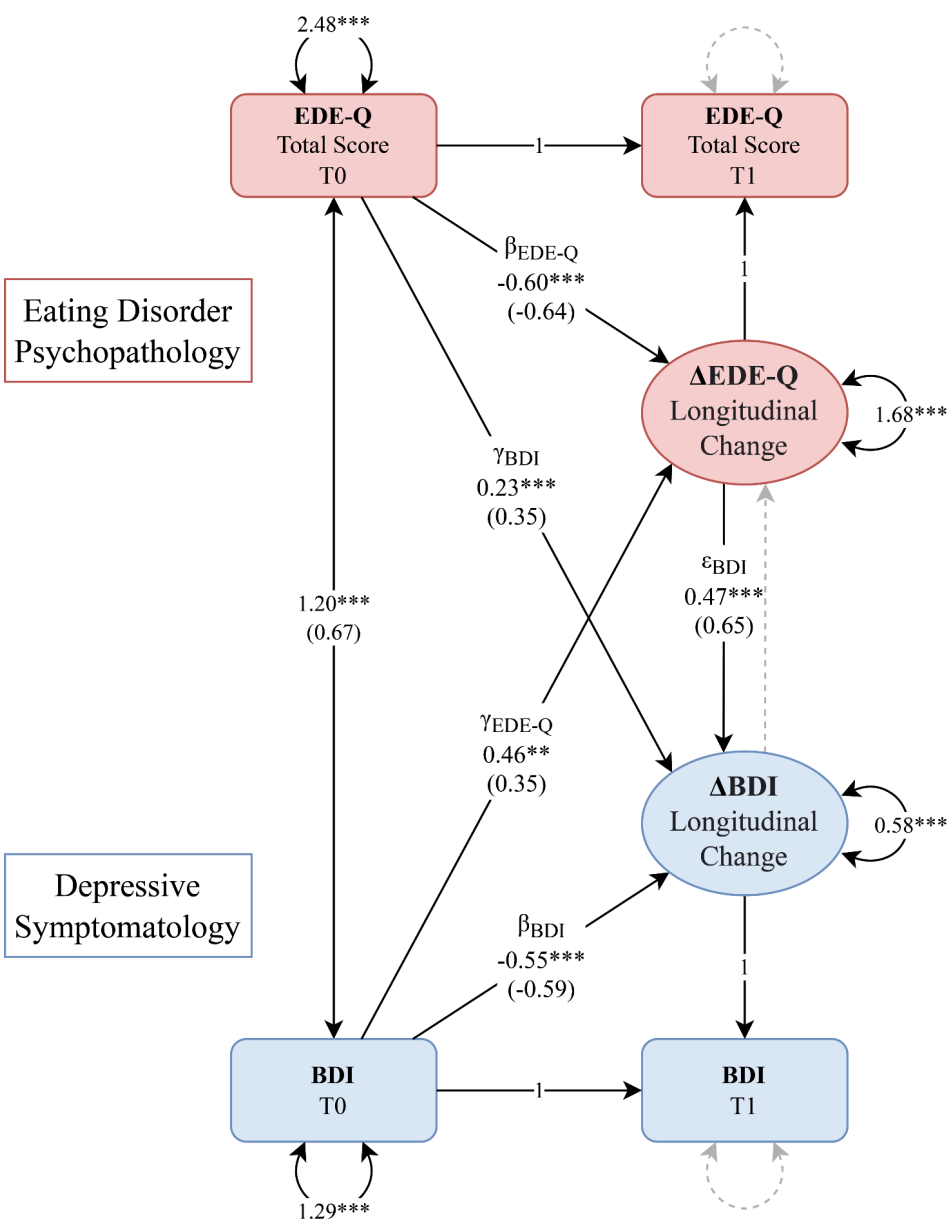
Bivariate latent change score (LCS) model of the associations between eating disorder psychopathology (in red) and depressive symptoms (in blue). Rectangles represent observed variables, and circles represent latent variables (LCSs). Regression effects and loadings are illustrated as single-headed arrows, labeled with their unstandardized and standardized (in parenthesis) coefficients. Effects or variances constrained to zero are illustrated as grey arrows, whereas those constrained to one are labeled as ‘1’. Double-headed arrows represent variances and covariances. ** *p* < 0.01, *** *p* < 0.001 BDI, Beck Depression Inventory; EDE-Q, Eating Disorder Examination Questionnaire.

Both autoregressive effects were statistically significant and negative, confirming the improvement over time in EDE-Q and BDI, proportional to the initial values (β_EDE-Q_ and β_BDI_ effects, Figure 1). The BDI_T0_→ΔEDE-Q cross-lagged effect (γ_EDE-Q_) was statistically significant, indicating that participants with higher levels of depressive symptoms at baseline showed a considerably worse outcome at follow-up in terms of ED psychopathology (Figure 1). Furthermore, patients with the most severe EDE-Q baseline scores showed less reduction in depressive symptoms at follow-up, as evidenced by the other cross-lagged effect (γ_BDI_) (Figure 1). In addition to the aforementioned cross-domain coupling effects, the variation in depressive symptoms at follow-up also significantly depended on the longitudinal amelioration of ED-specific psychopathology (ε_BDI_ effect, Figure 1), whereas the effect in the opposite direction was not supported by the model (Table 2).

## 4. Discussion

The present study attempted to clarify the complex interplay between depression and ED-specific psychopathology in patients with AN or BN treated with CBT-E. The main results were as follows: first, all psychopathological measures improved after treatment, including depressive symptoms; second, the augmentation of psychotherapeutic treatment with antidepressants did not modify the longitudinal trend of depression or ED psychopathology; third, greater depressive symptoms at baseline were associated with a poorer longitudinal trend of both ED psychopathology and depressive symptoms. Finally, for the first time, the bivariate LCS model showed that CBT-E determined an improvement in depression through the amelioration of ED psychopathology and not vice versa. 

The improvement in psychopathology, both general and ED-specific, was an expected result considering the well-known efficacy of CBT-E in the treatment of EDs [54,55]. In particular, the amelioration of depressive symptoms after CBT-E confirmed previous observations [27,56]. The fact that the augmentation of CBT-E with SSRI did not modify the longitudinal trend of either depression or ED-specific psychopathology, as demonstrated by the non-significant moderation analysis, confirmed that pharmacological treatment seems to have a limited role in the management of these disorders. In particular, this observation is in line with what was reported in a recent metanalysis about the ineffectiveness of antidepressants in the treatment of both affective problems and ED symptoms in patients with AN [23], and with the observation that, despite being effective in reducing bingeing–purging behaviors [57,58], the prescription of antidepressants in association with psychotherapy does not determine an amelioration of depressive symptoms in patients with BN [24]. Furthermore, the association between higher levels of depressive symptoms at baseline and poorer outcomes confirmed the hypothesis that depression represents an index of severity and a negative prognostic factor in the management of these disorders [5,6,7], highlighting the importance of gaining a deeper understanding of how treatments can effectively address this dimension.

Compared to the current scientific literature, the present study adopted LCS models, which allowed demonstrating that the improvement in depressive symptoms was predicted by the reduction in ED-specific psychopathology, in full accordance with the a priori hypothesis. In other words, the amelioration of ED symptoms predicted the improvement in depression and not vice versa. This result has significant clinical implications as, for the first time, it highlighted that addressing ED psychopathology as the primary target in these patients is of fundamental importance, independently of the presence of depressive symptoms, thus resolving the ambiguity about which symptom needs to be addressed first in the case of comorbidity. Indeed, the absence of a relationship between the improvement in depression and the amelioration of ED symptoms highlighted that targeting depressive symptoms without interrupting the vicious cycle of the ED might be counterproductive. This finding contrasts with the hypothesis according to which, in the case of severe depression, the initiation of CBT-E should be postponed until depressive symptoms improve [1]. Nevertheless, the result confirmed the importance of prioritizing ED symptoms, as stressed by several authors [25,26]. Furthermore, it is in line with the observations offered by Calugi et al. [27] about the improvement in both ED psychopathology and depression in patients with AN treated with CBT-E alone, also in the presence of clinical depression at baseline. It could be hypothesized that the reduction in the overinvestment in one’s capacity to control body shapes, weight, and nutrition in determining one’s self-esteem, and the consequent expansion of one’s horizon of values, may lead to a reduction in hopelessness increasing self-efficacy. Moreover, the recovery of the exploration of other areas of identity might reduce social isolation, restoring contacts with peers and allowing the achievement of life goals, with a consequent improvement in empowerment. Furthermore, a close relationship between ED psychopathology and the enactment of pathological eating behaviors has been well characterized, which may ultimately lead to severe states of malnutrition [1]. Therefore, it could be hypothesized that, in the present study, the erosion of ED psychopathology obtained through CBT-E might have had a role in determining an amelioration of depressive symptoms through the consequent improvement in nutritional status. Indeed, it is well known that many of the mood symptoms presented by patients with EDs overlap with signs of malnutrition, including fatigue, lack of energy, sleep disturbances, and scarce concentration [2]. Moreover, alterations in brain structure and neural networks were found in women with AN, and were positively correlated with malnutrition status [59,60]. Gauthier et al. also found that the reduction in depressive symptoms in patients with AN correlated with the increase in plasma tryptophan availability, obtained during refeeding [61].

The fact that only patients without a history of bipolar disorder or childhood trauma were included in the sample represents a strength of the present study. In fact, EDs are extremely heterogeneous, and, on the contrary, it is of primary importance to derive a better classification of mental disorders in order to possibly drive innovations in treatment [62]. For these reasons, it is necessary to provide explanatory models of the mechanisms of change that are characteristic of subpopulations who are as homogeneous as possible. This approach allows for a move in the direction of precision psychiatry, in full concordance with the novel frameworks of research domain criteria or hierarchical taxonomy of psychopathology [62,63], providing treatment strategies tailored to the characteristics of each individual. In particular, it is well known that patients with a history of trauma represent a different echo-phenotype with specific clinical and neurobiological characteristics [21,64,65,66,67,68], and, regarding the mechanisms of change induced by CBT-E in this population, a recent study showed that the amelioration of emotion dysregulation has a key role in determining the improvement in ED psychopathology in these patients [68].

These results should be interpreted in light of some limitations. First, the sample size was small, and only a single follow-up was performed. The present findings should be confirmed in larger studies, and further follow-up evaluations could be useful to verify that the observed improvements are maintained months after the conclusion of the treatment. Moreover, the reported findings are not generalizable to the populations of patients with AN and BN and a history of severe childhood trauma, severe depressive symptoms with suicidal ideation, or suffering from bipolar disorder. Further studies focused on these subgroups could show different longitudinal couplings between symptoms. Finally, data regarding the order of appearance of depressive and ED psychopathology were not available; as a result, insights into the order of onset could not be provided.

## 5. Conclusions

In conclusion, for the first time, this study provided an explicative model of the mechanism through which CBT-E determines an improvement in depressive symptoms in patients with AN or BN without a history of bipolarism or childhood trauma. In particular, it showed that the amelioration of depression in this subpopulation of patients depended on the improvement in ED symptoms, and not vice versa, providing clinicians with an important take-home message about the importance of addressing ED psychopathology as a primary target in these patients, independently from the presence of depressive symptoms, and resolving the ambiguity about which symptom needs to be addressed first in the case of comorbidity.

## Figures and Tables

**Table 1:** Characteristics of the sample at baseline and follow-up. Results of longitudinal analysis in patients are reported using age and BMI-adjusted unstandardized coefficients (time fixed effect).

	Baseline *T*0 (*n* = 157)	Follow-Up *T*1 (*n* = 121)	Time Effect (b)
Age (years)	25.52 ± 9.94	-	
Education (years)	12.88 ± 2.98	-	
Age of onset (years)	17.72 ± 5.19	-	
BMI (kg/m^2^)	19.33 ± 5.63	19.62 ± 4.63	0.62 *
EDE-Q Restraint	3.45 ± 1.87	2.25 ± 1.78	−1.31 ***
EDE-Q Eating Concern	2.97 ± 1.61	1.93 ± 1.60	−1.08 ***
EDE-Q Weight Concern	3.41 ± 1.72	2.63 ± 1.89	−0.84 ***
EDE-Q Shape Concern	3.87 ± 1.78	3.07 ± 1.94	−0.86 ***
EDE-Q Total Score	3.43 ± 1.58	2.47 ± 1.68	−1.03 ***
BDI	22.78 ± 11.41	13.85 ± 12.23	−9.28 ***
SCL-90-R GSI	1.52 ± 0.75	1.09 ± 0.77	−0.45 ***

* *p* < 0.05, *** *p* < 0.001 BDI, Beck Depression Inventory; BMI, Body Mass Index; EDE-Q, Eating Disorders Examination Questionnaire; SCL-90-R GSI, Symptom Checklist-90-Revised Global Severity Index.

**Table 2:** Results of nested model comparisons. The final model (reported in the first row) was used as a comparator for all analyses. For every nested model, the variation with respect to the final model is described in the first column. For every model, common goodness-of-fit measures are reported.

Model	χ^2^	DF	BIC	CFI	TLI	RMSEA	SRMR	Δχ^2^	Model
Final model	1.15	2	1711.43	1.00	1.01	0.000	0.033	-	Final model
Unconstrained models									
1→ΔEDE-Q	α_EDE-Q_ ≠ 0	0.86	1	1716.32	1.00	1.00	0.000	0.020	0.26
1→ΔBDI	α_BDI_ ≠ 0	0.26	1	1715.89	1.00	1.01	0.000	0.017	0.86
ΔBDI→ΔEDE-Q	ε_EDE-Q_ ≠ 0	0.58	1	1716.49	1.00	1.01	0.000	0.033	0.00
Constrained models									
EDE-Q_T0_⇻ΔEDE-Q	β_EDE-Q_ = 0	40.50 ***	3	1739.12	0.88	0.76	0.257	0.139	28.12 ***
BDI_T0_⇻ΔBDI	β_BDI_ = 0	52.15 ***	3	1740.62	0.87	0.75	0.265	0.114	49.18 ***
BDI_T0_⇻ΔEDE-Q	γ_EDE-Q_ = 0	12.45 **	3	1716.14	0.97	0.94	0.130	0.066	8.02 **
EDE-Q_T0_⇻ΔBDI	γ_BDI_ = 0	18.62 ***	3	1718.48	0.96	0.92	0.151	0.052	16.02 ***
ΔEDE-Q⇻ΔBDI	ε_BDI_ = 0	73.94 ***	3	1765.78	0.78	0.55	0.350	0.117	52.85 ***
cov_(EDE-Q T0, BDI T0)_ = 0		102.78 ***	3	1799.41	0.65	0.30	0.440	0.248	65.49 ***

** *p* < 0.01, *** *p* < 0.001 BDI, Beck Depression Inventory; BIC, Bayes Information Criterion; CFI, Comparative Fit Index; cov, covariance; DF, degrees of freedom; EDE-Q, Eating Disorders Examination Questionnaire; RMSEA, Root Mean Square Error of Approximation; SRMR, Standardized Root Mean Square Residual; TLI, Tucker–Lewis Index.

## Data Availability

The data that support the findings of this study are available from the corresponding author, upon reasonable request.

## Supplementary Materials

The following supporting information can be downloaded at: https://www.mdpi.com/article/10.3390/brainsci13040535/s1, Table S1: Characteristics of the sample at baseline and follow-up, divided by baseline diagnosis. Results of longitudinal analysis in patients are reported using age and BMI-adjusted unstandardized coefficients (time, group, and interaction fixed effects).
