# Supplementary material for: Longitudinal Coupling between Eating Disorder Psychopathology and Depression in Patients with Anorexia Nervosa and Bulimia Nervosa Treated with Enhanced Cognitive Behavior Therapy: A One-Year Follow-Up Study
Source: Brain Sci. 2023 Mar 24;13(4):535. doi: 10.3390/brainsci13040535 (PMC10136486; doi:10.3390/brainsci13040535)
Supplement: Supplementary file 1 [file brainsci-13-00535-s001.zip › brainsci-2280464-supplementary.pdf]

Longitudinal Coupling between Eating Disorder Psychopathology and Depression in Patients with Anorexia Nervosa and Bulimia Nervosa Treated with Enhanced Cognitive Behavior Therapy: A One-Year Follow-Up Study

Emanuele Cassioli, Eleonora Rossi, Michela Martelli, Francesca Arganini, Gabriele Giuranno, Serena Siviglia, Livio Tarchi, Marco Faldi, Giovanni Castellini * and Valdo Ricca

Psychiatry Unit, Department of Health Sciences, University of Florence, Largo Brambilla 3, 50100 Florence, Italy

Supplementary Materials

Supplementary Table S1. Characteristics of the sample at baseline and follow-up, divided by baseline diagnosis. Results of longitudinal analysis in patients are reported using age and BMI-adjusted unstandardized coefficients (time, group, and interaction fixed effects).

	**Anorexia Nervosa**		**Bulimia Nervosa**				
	Baseline *T0* (n = 92)	Follow-up *T1* (n = 73)	Baseline *T0* (n = 65)	Follow-up *T1* (n = 48)	Time Effect (b)	Group Effect (b)	Time*Group Interaction Effect (b)
Age (years)	24.41 ± 9.14	-	27.11 ± 10.86	-			
Education (years)	12.38 ± 3.09	-	13.52 ± 2.73	-			
Age of onset (years)	17.54 ± 4.03	-	17.97 ± 6.45	-			
EDE-Q Total Score	3.21 ± 1.64	2.29 ± 1.78	3.75 ± 1.44	2.73 ± 1.50	−1.10***	0.41	0.19
BDI	22.47 ± 11.59	14.00 ± 13.17	23.23 ± 11.24	13.62 ± 10.81	−9.10***	2.08	-0.36
SCL-90-R GSI	1.43 ± 0.78	1.07 ± 0.82	1.65 ± 0.68	1.13 ± 0.71	−0.42***	0.30	-0.06

* $p < 0.05$, ** $p < 0.01$, *** $p < 0.001$; BDI, Beck Depression Inventory; BMI, Body Mass Index; EDE-Q, Eating Disorders Examination Questionnaire; SCL-90-R GSI, Symptom Checklist-90-Revised Global Severity Index.
